# Assessment of the Patient Protection and Affordable Care Act’s Increase in Fees for Primary Care and Access to Care for Dual-Eligible Beneficiaries

**DOI:** 10.1001/jamanetworkopen.2020.33424

**Published:** 2021-01-21

**Authors:** Vicki Fung, Mary Price, Peter Hull, Benjamin Lê Cook, John Hsu, Joseph P. Newhouse

**Affiliations:** 1 Mongan Institute, Massachusetts General Hospital, Boston; 2 Department of Medicine, Harvard Medical School, Boston, Massachusetts; 3 Department of Economics, The University of Chicago, Chicago, Illinois; 4 National Bureau of Economic Research, Cambridge, Massachusetts; 5 Health Equity Research Lab, Cambridge Health Alliance, Harvard Medical School, Boston, Massachusetts; 6 Department of Psychiatry, Harvard Medical School, Boston, Massachusetts; 7 Department of Health Care Policy, Harvard Medical School, Boston, Massachusetts; 8 Department of Health Policy and Management, Harvard T.H. Chan School of Public Health, Boston, Massachusetts; 9 Harvard Kennedy School, Cambridge, Massachusetts

## Abstract

**Importance:**

The Patient Protection and Affordable Care Act (ACA) temporarily increased primary care practitioners’ (PCP) Medicaid fees to that of Medicare for 2013 to 2014 (fee bump) to help accommodate potential increases in demand for care with ACA coverage expansion. This also increased fees for PCPs treating dual-eligible Medicare and Medicaid beneficiaries in many states and eliminated payment differentials for dual-eligible vs non–dual-eligible Medicare beneficiaries that could limit access to care.

**Objective:**

To examine the association between the ACA fee bump and primary care visits for dual-eligible Medicare and Medicaid beneficiaries.

**Design, Setting, and Participants:**

This cohort study used a difference-in-difference design and Medicare claims data from 2012 to 2016 to compare changes in visit rates for full-subsidy dual-eligible Medicare and Medicaid beneficiaries vs non–dual-eligible Medicare beneficiaries with low income whose fees did not change. Changes were examined overall and separately in states with temporary, extended, or minimal fee increases for dual-eligible vs non–dual-eligible beneficiaries in 2013 to 2014 (mandatory bump) and 2015 to 2016 (postbump or bump extension) vs 2012 (prebump). The study used linear regression models with beneficiary fixed effects, adjusting for time-changing area and beneficiary characteristics. Statistical analysis was performed from February 2018 to November 2019.

**Exposure:**

ACA-mandated Medicaid fee bump.

**Main Outcomes and Measures:**

Primary care visits per 100 beneficiaries overall and visits billed by physicians vs nurse practitioners and physician assistants.

**Results:**

The study included 3 052 044 dual-eligible and non–dual-eligible beneficiaries in 2012; 1 516 534 (49.7%) were aged 65 years or younger, 1 797 556 (58.9%) were women, and 1 754 626 (57.5%) had non-Hispanic White race/ethnicity. Overall primary care visit rates for dual-eligible beneficiaries were unchanged or decreased slightly relative to non–dual-eligible beneficiaries during the fee bump (2013-2014) and the postbump or bump extension period (2015-2016) vs baseline. Compared with non–dual-eligible beneficiaries, visit rates with primary care physicians declined more uniformly for dual-eligible beneficiaries across state groups and time periods (difference-in-difference: −0.37 [95% CI, −0.43 to −0.32] visits per 100 beneficiaries in 2013-2014 vs 2012; *P* < .001; and difference-in-difference: −0.62 [95% CI, −0.68 to −0.56] visits per 100 beneficiaries in 2015-2016 vs 2012; *P* < .001), whereas visits with nurse practitioners and physician assistants increased over time (difference-in-difference: 0.11 [95% CI, 0.08 to 0.14] visits per 100 beneficiaries in 2013-2014 vs 2012; *P* < .001; and difference-in-difference: 0.46 [95% CI, 0.43 to 0.50] visits per 100 beneficiaries in 2015-2016 vs 2012; *P* < .001). These changes, however, were not associated with the timing of the payment changes.

**Conclusions and Relevance:**

The ACA fee bump was not associated with increases in primary care visits for dual-eligible Medicare and Medicaid beneficiaries. Visits for dual-eligible beneficiaries with primary care physicians decreased after the ACA, a decrease that was partially offset by increases in visits with nonphysician clinicians.

## Key Points

**Question:**

Was the Affordable Care Act’s increase in Medicaid fees to Medicare levels for primary care practitioners (fee bump) associated with increases in visits for dual-eligible Medicare and Medicaid beneficiaries?

**Findings:**

In this cohort study of 3 052 044 Medicare beneficiaries, primary care visit rates for dual-eligible Medicare and Medicaid beneficiaries did not increase during years of the fee bump compared with Medicare beneficiaries with low income whose fees did not change. Decreases in relative visit rates with primary care physicians were partially offset by increases in visits with nurse practitioners and physician assistants.

**Meaning:**

In this study, the policy eliminated payment differentials for dual-eligible vs non–dual-eligible Medicare beneficiaries, but it was not associated with gains in primary care use for dual-eligible beneficiaries.

## Teaser

This cohort study evaluates whether the Affordable Care Act’s increase in Medicaid fees to Medicare levels for primary care practitioners was associated with increases in primary care visits for dual-eligible Medicare and Medicaid beneficiaries.

## Introduction

Dual-eligible Medicare and Medicaid beneficiaries account for more than one-third of Medicare and Medicaid spending, and frequently have multiple chronic conditions, severe mental illness, and disability.^1,2^ Dual-eligible beneficiaries are more likely to have potentially preventable hospital admissions compared with non–dual-eligible beneficiaries,^3^ and reports suggest that patients with dual eligibility often face problems accessing care, in part because many practitioners are reimbursed less for dual-eligible Medicare beneficiaries.^4,5,6,7^ The Patient Protection and Affordable Care Act (ACA) required that states increase Medicaid payments for primary care practitioners (PCPs) to Medicare levels in 2013 and 2014 for certain services (referred to as the *fee bump*), which also increased fees for PCPs treating dual-elegible patients in most states.

Medicare is the primary payer for primary care services for individuals with dual eligibility, and Medicaid provides wraparound coverage that pays the Medicare cost-sharing for qualified beneficiaries. However, many states cap Medicaid reimbursement for Medicare cost-sharing at the Medicaid rate (known as lesser-of policies), such that health care practitioners frequently receive partial or no reimbursement for the standard 20% coinsurance for dual-eligible patients.^6,8^ A recent analysis found that the number of states capping dual reimbursement increased from 36 to 42 between 2004 and 2018.^9^ Federal law prohibits health care practitioners from directly billing dual-eligible beneficiaries for the uncovered coinsurance amount, although some improper billing occurs, which can further impede access to care for these patients.^10^

The ACA fee bump temporarily increased primary care Medicaid payments to 100% of the Medicare rate to address potential barriers to care associated with low Medicaid fees, which are often identified by physicians as a deterrent to accepting Medicaid patients.^11,12,13,14^ In 2012, the range of Medicaid-to-Medicare payment ratios ranged from 33% to 135%, with a national average of 59%.^15^ Existing studies on the impact of the ACA fee bump focus on Medicaid-only enrollees and have not found increases in PCPs’ participation in Medicaid, although 1 study found increases in appointment availability for Medicaid vs commercial enrollees during the fee bump.^16,17,18,19^ These studies have not examined changes in beneficiary use associated with the fee bump, and those examining PCPs’ Medicaid participation exclude nurse practitioners (NPs) and physician assistants (PAs), who are increasingly providing primary care, especially in rural and low-income areas where Medicare beneficiaries are more likely to be dual.^20,21^ NPs and PAs were eligible for the fee bump if working under the supervision of a physician.

Although the magnitude of the payment increases was considerably smaller, on average, for dual-eligible vs Medicaid-only enrollees, the effects of the fee bump could differ for those with dual eligibility. There were delays in implementing the policy in many states, with greater challenges within the context of Medicaid managed care plans with capitated or bundled payment systems, which could have contributed to its muted effect in the Medicaid population.^17,22,23^ PCPs treating dual-eligible patients were more likely to receive the fee bump through fee-for-service payments because these patients were less likely to be in managed care plans compared with Medicaid-only enrollees (eg, 24% of dual eligible patients vs 67% of Medicaid enrollees in 2016, although there was wide variation across states).^24,25^ Dual beneficiaries could also have been more likely to be linked to a PCP at baseline and seek care because of their higher levels of clinical need, especially compared with the Medicaid expansion population of adults with low income and no disability. Examining the impact of the ACA fee bump on dual-eligible individuals is important for informing ongoing state policy changes regarding dual payment policy.

In 2015, 34 states decreased PCP payments to pre-2013 rates after expiration of federal funding, while 16 states continued the fee bump using state funds.^26^ In this study, we examined changes in primary care visits for dual-eligible patients overall, and differences for those living in states with temporary vs extended vs minimal fee increases, and in states that expanded and did not expand Medicaid.

## Methods

### Study Data and Population

This cohort study was approved for the research and use of health and medical records with a waiver of informed consent by the Mass General Brigham institutional review board. This study follows the Strengthening the Reporting of Observational Studies in Epidemiology (STROBE) reporting guideline.

We used a difference-in-difference approach to assess the fee bump and its associations with access to primary care for dual-eligible Medicare and Medicaid beneficiaries. We identified dual-eligible beneficiaries who received assistance from Medicaid to cover their Medicare cost-sharing, including Qualified Medicare Beneficiaries (QMB) and other full-benefit dual-eligible patients. The federal income limit for QMB eligibility is 100% of the federal poverty level (FPL). To improve the comparability of our control group, we focused on Medicare beneficiaries with incomes just above the thresholds for cost-sharing subsidies. We identified this group by their receipt of other low-income subsidies, including non-QMB partial dual-eligible beneficiaries and non–dual-eligible beneficiaries who received Full Part D low-income subsidies. The federal income limit for these programs is 135% FPL. For simplicity, we refer to the low-income control group not exposed to the fee change as non–dual-eligible patients.

We used fee-for-service Medicare claims data for a 50% sample of the beneficiaries with low income from 2012 to 2016. Individuals could enter the sample (eg, if they newly receive subsidies or enter Medicare) or exit (because of death, switching to Medicare Advantage, or no longer receiving subsidies) in each month of the study; a mean of 11.5% of beneficiaries were new and 12.8% exited in each year. We excluded beneficiaries who were institutionalized.^27^

Because Medicare covers 80% of the total cost of a visit, for dual-eligible patients in most states with lesser-of dual reimbursement policies, the fee bump increased PCP payments from 80% to 100% of the Medicare fee. For example, if the Medicare fee for a visit is $100, Medicare covers $80, non–dual-eligible beneficiaries have a $20 coinsurance payment, and Medicaid covers the coinsurance payment for dual-eligible patients. In the absence of the fee bump, if a state uses a lesser-of reimbursement policy and the Medicaid rate for the visit is $80 or less, PCPs do not receive any reimbursement for the $20 coinsurance. Similarly, for states with Medicaid rates between $80 and $100 (eg, $95), PCPs can be reimbursed up to the Medicaid rate (eg, $15). In contrast, for states with full reimbursement policies or Medicaid payment rates at or above Medicare rates (eg, during the fee bump), PCPs can be reimbursed for the full coinsurance amount.

We grouped beneficiaries by the magnitude and duration of the fee bump on the basis of their state (eTable in the Supplement). We classified 28 states as having temporary fee bumps if their Medicaid-to-Medicare fee ratios were less than 90% in 2012, they used a lesser-of dual reimbursement policy, and they did not extend the fee bump beyond the federal financing period of 2013 to 2014. We classified 6 states as having extended fee bumps, meaning that they met the first 2 criteria, but also extended the fee bump using state funds after 2014. Finally, we classified 16 states as having minimal or no changes in fees because the state had a full dual reimbursement policy or high baseline Medicaid-to-Medicare fee ratios in 2012 (the lowest in these states was 94%).

### Outcome

The fee bump applied to evaluation and management (E&M) visits with *Current Procedural Terminology *(*CPT*) codes 99201 to 99499 and vaccine administration and counseling (*CPT *codes 90460, 90461, and 90471-90473). Eligible clinicians included those practicing in primary care with a specialty in family medicine, general internal medicine, or pediatric medicine, or a subspecialty recognized by certain physicians’ associations. We included physicians with primary taxonomy codes in the National Provider Identifier file of general medicine, family medicine, or internal medicine, and clinicians with primary care NP or PA taxonomy codes.

We examined changes in all outpatient visits in each study month for dual-eligible beneficiaries and non–dual-eligible beneficiaries. We present the findings focused on all visits to outpatient service settings instead of only E&M visits because of potential confounding associated with an unrelated 2013 coding change for mental health services that converted mental health–specific codes to E&M *CPT* codes. Because visits with NPs and PAs are difficult to distinguish in claims data (these visits are often billed by a physician because of payment differentials),^28,29^ our primary findings focus on changes in aggregate primary care visits to all clinician types. In secondary analyses, we examined changes in visit rates billed by physicians vs NPs and PAs.

### Statistical Analysis

Our primary unit of analysis was the beneficiary-month. We used a difference-in-difference approach to estimate changes in primary care visits for dual-eligible vs non–dual-eligible beneficiaries before and after the fee bump. We used linear regression models to estimate the changes in monthly visit rates in years of the fee bump (2013-2014) vs the year before the bump (2012) and years after bump expiration or extended fee bump (2015-2016) vs 2012. We stratified models by state Medicaid expansion status (as of the end of 2014) and state policy groups to assess differences in states with and without fee bumps and with temporary vs extended policy changes. Similar to Mulcahy et al,^19^ we also present changes in visit rates by individual state.

The models included beneficiary-level fixed effects to account for potential time-stable unmeasured confounders. We also adjusted for annually changing measures of beneficiaries’ individual-level Centers for Medicare & Medicaid Services Hierarchical Condition Categories comorbidity scores and indicators for whether they were aligned to an accountable care organization. Because insurance coverage expansion could impact the local PCP capacity, we adjusted for the percentage of residents within each beneficiary’s county with insurance coverage in each year using the American Community Survey (ie, 1-year estimates, 2012-2016). Finally, because temporal trends could have varied by state because of other policy changes, we also adjusted for annual and monthly trends at the state level.

In sensitivity analyses, we excluded 8 states that implemented dual demonstration programs during the study period that encouraged dual beneficiaries to enroll in capitated Medicare Advantage plans. We also examined changes in visits to federally qualified health centers (FQHCs) and rural health centers. The fee bump did not apply to these clinics; however, the ACA included additional funding for FQHCs. Thus, we assessed whether changes in our outcomes were due to shifts in site of care associated with concurrent, but separate policy changes. All statistical analyses were performed using SAS statistical software version 9.4 (SAS Institute). Statistical significance was set at *P* < .05 and all tests were 2-sided. The analysis was performed from February 2018 to November 2019.

## Results

Our study included 3 052 044 dual-eligible and non–dual-eligible beneficiaries in 2012; 1 516 534 (49.7%) were aged 65 years or younger, 1 797 556 (58.9%) were women, and 1 754 626 (57.5%) had non-Hispanic White race/ethnicity (Table 1). Dual-eligible beneficiaries were more likely to be of non-White race/ethnicity and had higher mean comorbidity scores vs non–dual-eligible beneficiaries. Beneficiaries’ demographic characteristics were similar in 2016.

**Table 1.** Study Population Characteristics in 2012 and 2016

Characteristic	Beneficiaries, No. (%)			
	2012		2016	
	Full subsidy dual-eligible^a^ (n = 2 247 054)	Non–dual-eligible^b^ (n = 804 990)	Full subsidy dual-eligible (n = 2 148 507)	Non–dual-eligible (n = 754 845)
Age group, y				
<65	1 155 147 (51.4)	361 387 (44.9)	1 122 812 (52.3)	345 500 (45.8)
65-74	539 522 (24.0)	226 218 (28.1)	541 646 (25.2)	223 840 (29.7)
75-84	378 242 (16.8)	149 021 (18.5)	325 718 (15.2)	127 072 (16.8)
≥85	174 143 (7.7)	68 364 (8.5)	158 331 (7.4)	58 433 (7.7)
Sex				
Female	1 339 600 (59.6)	457 956 (56.9)	1 256 986 (58.5)	422 215 (55.9)
Male	907 454 (40.4)	327 034 (43.1)	891 521 (41.5)	332 630 (44.1)
Original reason for entitlement				
Age	852 061 (37.9)	337 746 (42.0)	779 374 (36.3)	295 818 (39.2)
Disability	1 350 132 (60.1)	450 698 (56.0)	1 325 722 (61.7)	442 944 (58.7)
ESKD	16 700 (0.7)	3104 (0.4)	21 911 (1.0)	5481 (0.7)
ESKD and disability	28 161 (1.3)	13 442 (1.7)	21 500 (1.0)	10 602 (1.4)
Race/ethnicity				
American Indian or Alaskan Native	27 538 (1.2)	7871 (1.0)	29 048 (1.4)	8290 (1.1)
Asian or Pacific Islander	149 313 (6.6)	18 776 (2.3)	146 405 (6.8)	16 902 (2.2)
Black	455 587 (20.3)	160 779 (20.0)	413 301 (19.2)	146 305 (19.4)
Hispanic	359 267 (16.0)	80 589 (10.0)	332 509 (15.5)	74 413 (9.9)
Non-Hispanic White	1 224 850 (54.5)	529 776 (65.8)	1 187 058 (55.3)	499 831 (66.2)
Other or unknown	30 499 (1.4)	7199 (0.9)	40 186 (1.9)	9104 (1.2)
HCC Comorbidity Score, mean (SD)	1.37 (1.22)	1.27 (1.15)	1.28 (1.13)	1.19 (1.07)
Aligned to an ACO	57 741 (2.6)	10 731 (1.3)	423 567 (19.7)	155 444 (20.6)
Live in Medicaid expansion state	1 283 331 (57.1)	348 821 (43.3)	1 256 953 (58.5)	353 819 (46.9)
Live in state with fee bump				
Temporary	1 751 490 (77.9)	610 378 (75.8)	1 694 716 (78.9)	554 289 (73.4)
Extended	159 508 (7.1)	60 990 (7.6)	151 658 (7.1)	60 221 (8.0)
No or minimal	336 056 (15.0)	133 622 (16.6)	302 133 (14.1)	140 335 (18.6)

Abbreviations: ACO, Accountable Care Organization; ESKD, end stage kidney disease; FPL, federal poverty level; HCC, Hierarchical Condition Categories.

^a^ <100% FPL.

^b^ 100%-135% FPL.

### Changes in Primary Care Visits After the Fee Bump

Dual-eligible beneficiaries had a mean of 41.6 primary care visits per 100 beneficiaries per month in the baseline year, 2012, compared with 36.6 visits per 100 beneficiaries for non–dual-eligible beneficiaries (Table 2). Adjusted visit rates increased slightly in 2013 to 2014 and 2015 to 2016 for both dual-eligible and non–dual-eligible beneficiaries vs 2012. The fee bump was associated with a small decline in relative visit rates for dual-eligible beneficiaries (difference-in-difference: −0.26 [95% CI, −0.33 to −0.20] visits per 100 beneficiaries in 2013-2014 vs 2012; *P* < .001) (Table 2). In the postpolicy periods, there were relative decreases in visits for dual-eligible vs non–dual-eligible beneficiaries billed by primary care physicians (difference-in-difference: −0.37 [95% CI, −0.43 to −0.32] visits per 100 beneficiaries in 2013-2014 vs 2012; *P* < .001; and difference-in-difference: −0.62 [95% CI, −0.68 to −0.56] visits per 100 beneficiaries in 2015-2016 vs 2012; *P* < .001) and increases in visits billed by NPs and PAs (difference-in-difference: 0.11 [95% CI, 0.08 to 0.14] visits per 100 beneficiaries in 2013-2014 vs 2012; *P* < .001; and difference-in-difference: 0.46 [95% CI, 0.43 to 0.50] visits per 100 beneficiaries in 2015-2016 vs 2012; *P* < .001).

**Table 2.** Monthly Primary Care Visit Rates Per 100 Beneficiaries for Dual-Eligible and Non–dual-eligible Beneficiaries^a^

Visit type	Visit rate/100 beneficiaries						Difference-in-difference rate for dual-eligible vs non–dual-eligible (95% CI)	
	Dual-eligible beneficiaries			Non–dual-eligible Medicare beneficiaries				
	Prebump (2012)	Bump (2013-2014)	Postbump or extension (2015-2016)	2012	2013-2014	2015-2016	2013-2014 vs 2012	2015-2016 vs 2012
All primary care visits	41.6	43.0	42.6	36.6	38.3	37.7	−0.26 (−0.33 to −0.20)^b^	−0.15 (−0.22 to −0.08)^b^
Billed by physician	34.3	34.5	31.0	30.4	31.0	27.7	−0.37 (−0.43 to −0.32)^b^	−0.62 (−0.68 to −0.56)^b^
Billed by NPs and PAs	7.3	8.5	11.6	6.2	7.3	10.1	0.11 (0.08 to 0.14)^b^	0.46 (0.43 to 0.50)^b^

Abbreviations: NP, nurse practitioner; PA, physician assistant.

^a^ Adjusted visit rates estimated from a linear model with a person-level fixed effect and the 2012 non–dual-eligible visit rates. Models also adjust for the percentage of residents in the county insured in each year, individual-level Hierarchical Condition Categories scores in each year, an annual flag for Accountable Care Organization alignment, and state-policy period and state-month fixed effects.

^b^ *P* < .001.

### Differences Across States by Fee Bump Duration and Medicaid Expansion

Among Medicaid expansion states, there were relative decreases in dual-eligible visit rates during the fee bump implementation and expiration periods in states with temporary fee bumps (eg, difference-in-difference: −0.40 [95% CI, −0.51 to −0.30] visits per 100 beneficiaries in 2013-2014 vs 2012) and minimal fee changes (difference-in-difference: −0.25 [95% CI, −0.48 to −0.03] visits per 100 beneficiaries). In states that expanded Medicaid with extended fee increases, relative dual-eligible visit rates did not change significantly. Visit rates for dual-eligible vs non–dual-eligible beneficiaries in non-Medicaid expansion states largely did not change across states with varying fee increases.

**Figure 1.**
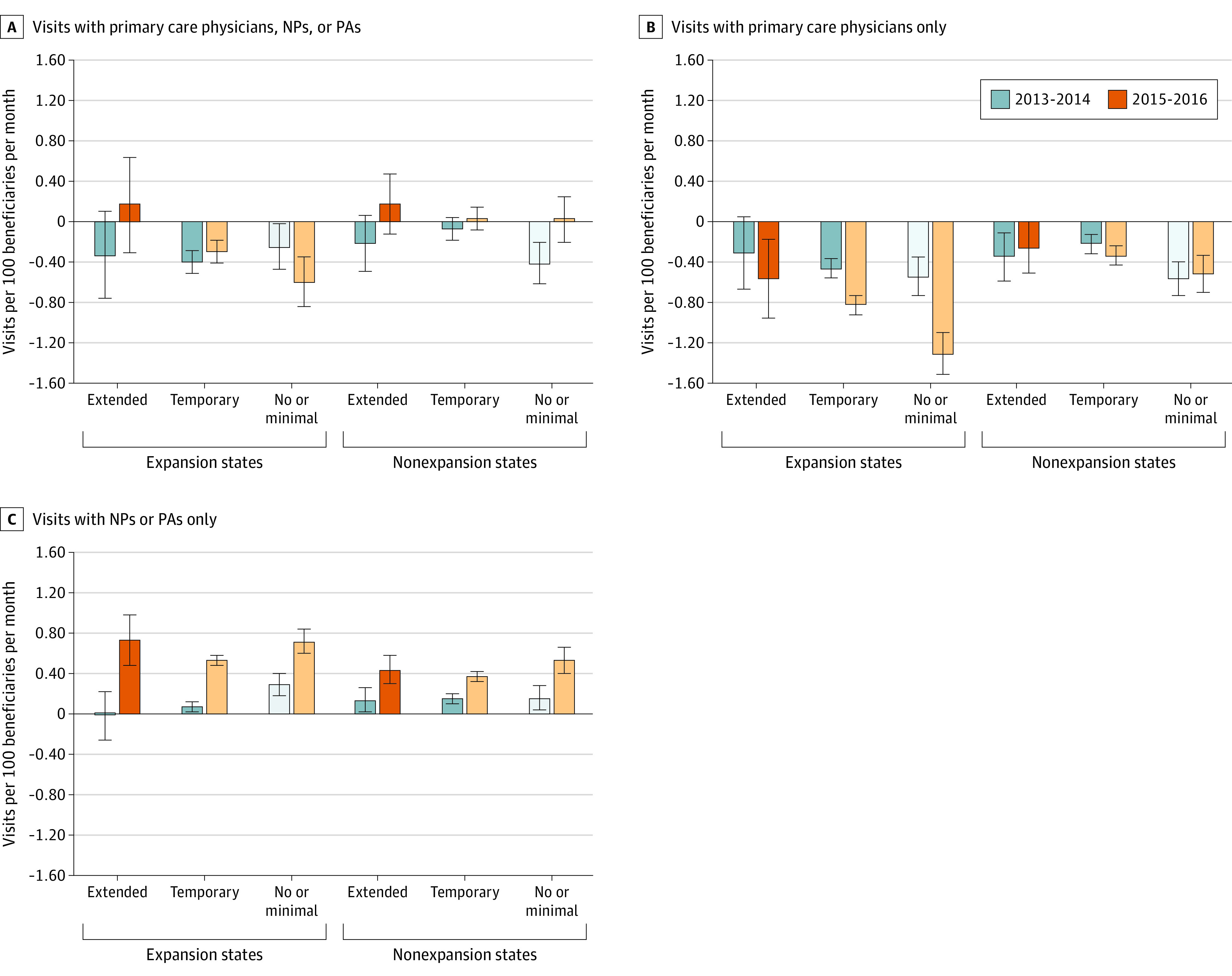
Changes in Monthly Primary Care Visit Rates for Dual-Eligible vs Non–dual-eligible Beneficiaries Compared in Years With the Fee Bump and Postbump vs Prebump (2012) per 100 Beneficiaries Dark blue and dark orange bars represent years in which the fee bump was active; light blue and light orange bars represent years in which the fee bump was expired in states with temporary fee bumps, or there was no or minimal fee change. Models also adjust for the percentage of residents in the county insured in each year, individual-level Hierarchical Condition Categories scores in each year, an annual flag for Accountable Care Organization alignment, and state-year and state-month fixed effects.

In secondary analyses, relative visit rates declined more uniformly across state groups and time periods for visits billed by primary care physicians, whereas visits billed by NPs and PAs increased over time for dual-eligible vs non–dual-eligible beneficiaries (Figures 1B and 1C). There were no significant changes in the proportion of dual-eligible vs non–dual-eligible individuals that had at least 1 annual primary care visit, with the exception of a 0.36 (95% CI, 0.15 to 0.57) increase in 2015 to 2016 vs 2012 in states that expanded Medicaid with temporary fee increases (eFigure 1 in the Supplement). Our findings were robust to sensitivity analyses and there were no relative increases in visits to FQHCs or RHCs for dual-eligible individuals in the postpolicy period (eFigure 2 in the Supplement).

### State Variation

**Figure 2.**
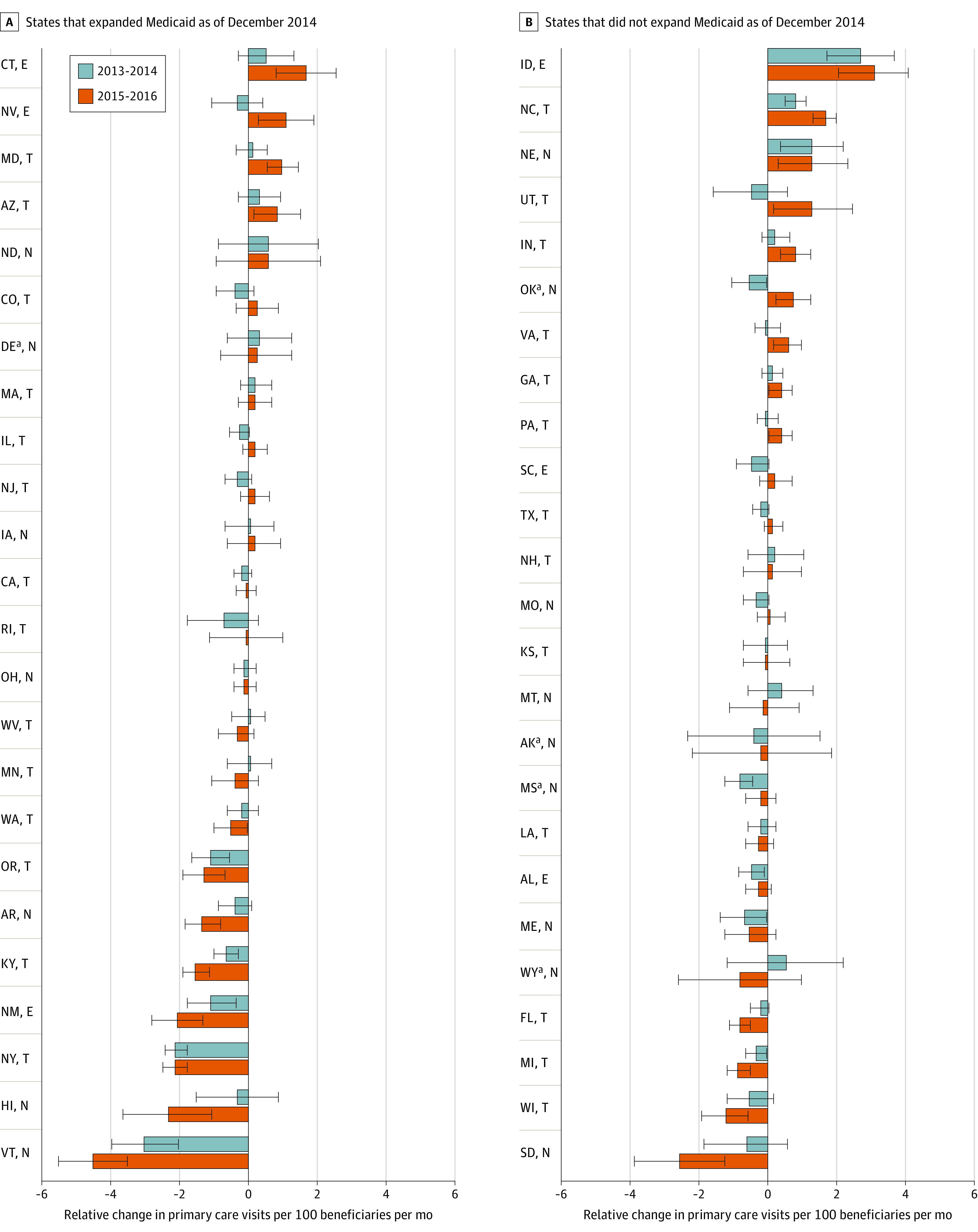
State-Level Changes in Monthly Primary Care Visits for Dual-Eligible vs Non–dual-eligible Beneficiaries Compared With 2012 Bars represent 95% CIs; not adjusted for multiple comparisons. E indicates extended fee bump; N, no or minimal fee change; and T, temporary fee bump (2013-2014 only). ^a^States with high baseline Medicaid-Medicare payment ratios (not full payment states).

There was substantial heterogeneity at the state level in changes in visit rates for dual-eligible vs non–dual-eligible beneficiaries (Figure 2). In 2015 to 2016 vs 2012, relative visit rates for dual-eligible patients increased significantly in 4 expansion and 9 nonexpansion states and decreased significantly in 8 expansion and 3 nonexpansion states. Postpolicy period changes were generally in the same direction within each state across the years studied, with larger changes in the later time period.

## Discussion

We assessed whether the ACA fee bump that was intended to improve PCP availability for populations with low income was associated with increases in primary care visits among dual-eligible beneficiaries. Although the policy increased fees for PCPs treating dual-eligible patients by up to 25% in many states, we did not find temporary or sustained increases in primary care visits for dual-eligible beneficiaries living in states with fee increases vs comparable non–dual-eligible beneficiaries, on average. This result is consistent with other studies that have examined the impact of the fee bump on changes in practitioners’ Medicaid participation for Medicaid-only enrollees.^17,18,19^

These studies and others have noted features of the policy that could have limited its effects, including its limited duration and problems in many states with the initial implementation, including slow announcement of the changes, limited practitioner knowledge of the policy or how to self-attest for program eligibility, and delayed reimbursements.^17,22,23,30^ Although the fee bump could have helped PCPs recoup the Part B coinsurance for dual-eligible patients, most would be required to bill Medicaid separately for this increment. Reports suggest that this process is administratively cumbersome in many states, especially for practitioners who were not already enrolled in Medicaid in some states.^4,31^

Dual-eligible patients have high levels of clinical need and spending, but there is limited information on how payment policy affects access to care for these individuals. At the same time, there have been ongoing changes in payment policy for dual-eligible patients, with more states capping reimbursement.^9^ In contrast to our study, a prior study^32^ found a positive association between full vs lesser-of reimbursement policy and the likelihood that dual-eligible patients had an outpatient visit. Practitioners could be more likely to change their behavior in response to state changes in dual payment policy if these are viewed as less temporary in nature than the ACA fee bump. We found a slightly positive uptick in mean visits for dual-eligible patients in the extension period among states that continued the fee bump beyond 2014, but these estimates were not statistically significant. This could signal that fee increases are associated with increased access when implemented longer term, but that these associations also take time to manifest. Additional years of follow-up are needed to confirm this finding because many state decisions to extend the policy were not clarified until close to the end of the initial implementation period.^33^ In addition, some state policy makers noted that they continued the fee bump because of other perceived benefits, including improving relationships and goodwill between PCPs and Medicaid.^22^

The broader literature examining the impact of Medicaid fee changes on use is mixed and highlights the potential influence of the local market and policy context.^34^ We found wide variation in changes in visit rates for dual-eligible beneficiaries across states. Local shifts in the primary care workforce and practice patterns could be particularly important. We found relative increases in visit rates for dual-eligible patients that were billed by NPs and PAs over the study period, which helped to offset more consistent decreases in visits billed by primary care physicians. It is possible that these findings could reflect changes in billing vs care patterns; however, our study design focused on comparisons of dual-eligible vs non–dual-eligible beneficiaries living in the same state to account for other state policy changes (eg, scope of practice laws) that could influence billing practices. In addition, these findings are consistent with evidence of increasing supply of nonphysician primary care practitioners, especially in lower-income and rural areas, changes in primary care team practice, and trends of declining caseloads of dual-eligible patients among primary care physicians.^20,21,35,36^ Nevertheless, we did not find that relative increases in visits with NPs or PAs were associated with the timing of payment changes, and qualitative studies suggest that some NPs and PAs, especially those practicing independently, had difficulties applying for the payment increase.^23^ Thus, these findings could reflect general trends that are not directly associated with the fee bump.

### Limitations

This study has limitations. First, it was nonrandomized and there could be residual confounding. We attempted to reduce potential unmeasured differences between our comparison groups by limiting the control group to Medicare beneficiaries who qualified for other low-income subsidies and were just above the income thresholds for full subsidy dual eligibility. Our analyses included beneficiary fixed effects to address potential unmeasured time-stable differences between our comparison groups and various time-varying controls. However, if changes in Medicaid provisions (eg, Medicaid managed care penetration or adoption of alternative payment models in Medicaid) differentially affected visit rates for dual-eligible vs non–dual-eligible beneficiaries, this could introduce bias. Second, we did not have information on whether practitioners actually received the fee bump. However, we focused on the 3 main primary care specialties that were most likely to be eligible for the fee bump. NPs and PAs had to be practicing under the supervision of a physician (as attested to by a physician) to be eligible, although we did not have this specific information. In addition, we were unable to examine changes in practitioner’s panel composition across payers to assess whether declines in visits were the result of ACA-related crowd-out (ie, whether practitioners were treating more non–dual-eligible beneficiaries, leading to decreased visit capacity for dual-eligible beneficiaries).

## Conclusions

This cohort study did not find evidence that the fee bump was associated with increased visits to primary care for dual-eligible Medicare and Medicaid beneficiaries. Visits with independent NPs and PAs partially offset declines in visits with physicians after the ACA implementation, underscoring the importance of nonphysician clinicians in the primary care workforce, especially for underserved populations.

## Notes

Supplement. **eTable.** Summary of State Medicaid Policies **eFigure 1.** Changes in the Percent of Dual-Eligible vs Non-Dual-Eligible Beneficiaries With at Least 1 Annual Visit With a Primary Care Provider Before and After the Fee Bump **eFigure 2.** Change in Monthly Visits to Federally Qualified Health Centers and Rural Health Centers for Dual vs Non-Dual Beneficiaries Before and After the Fee Bump **eReferences.**
