# Supplementary material for: Assessment of the Patient Protection and Affordable Care Act’s Increase in Fees for Primary Care and Access to Care for Dual-Eligible Beneficiaries
Source: JAMA Netw Open. 2021 Jan 21;4(1):e2033424. doi: 10.1001/jamanetworkopen.2020.33424 (PMC7821030; doi:10.1001/jamanetworkopen.2020.33424)
Supplement: Supplement. — eTable. Summary of State Medicaid Policies eFigure 1. Changes in the Percent of Dual-Eligible vs Non-Dual-Eligible Beneficiaries With at Least 1 Annual Visit With a Primary Care Provider Before and After the Fee Bump eFigure 2. Change in Monthly Visits to Federally Qualified Health Centers and Rural Health Centers for Dual vs Non-Dual Beneficiaries Before and After the Fee Bump eReferences. [file jamanetwopen-e2033424-s001.pdf]

Supplemental Online Content

Fung V, Price M, Hull P, Cook BL, Hsu J, Newhouse JP. Assessment of the Patient Protection and Affordable Care Act's increase in fees for primary care and access to care for dual-eligible beneficiaries. *JAMA Netw Open*. 2021;4(1):e2033424. doi:10.1001/jamanetworkopen.2020.33424

eTable. Summary of State Medicaid Policies
eFigure 1. Changes in the Percent of Dual-Eligible vs Non-Dual-Eligible Beneficiaries With at Least 1 Annual Visit With a Primary Care Provider Before and After the Fee Bump
eFigure 2. Change in Monthly Visits to Federally Qualified Health Centers and Rural Health Centers for Dual vs Non-Dual Beneficiaries Before and After the Fee Bump
eReferences.

This supplemental material has been provided by the authors to give readers additional information about their work.

© 2021 Fung V et al. *JAMA Network Open.*

eTable. Summary of State Medicaid Policies

	Medicaid to Medicare Fee Index							
**State**	**2012[1]**	**2014[2]**	**2016[3]**	**Medicaid Expansion as of Dec 31, 2014**	**Dual-eligible Medicare cost-sharing reimbursement policy as of 2015**	**Fee bump status as of 2015**	**State group for analyses**	**Dual demo enroll start date**
Alabama	0.7	0.65	1		Lesser-of	Full	Extended	
Alaska	1.27	1.28	1.27	[a]	Lesser-of	No	No/minimal	
Arizona	0.75	0.73	0.73	Y	Lesser-of	No	Temporary	
Arkansas	0.7	0.68	0.65	Y	Full	No	No/minimal	
California	0.43	0.42	0.41	Y	Lesser-of	No	Temporary	4/1/2014
Colorado	0.74	0.73	0.84	Y	Lesser-of	No	Temporary	
Connecticut	0.71	0.78	0.76	Y	Lesser-of	Full [b]	Extended	
Delaware	0.98	0.98	0.99	Y	Lesser-of	No	No/minimal	
DC	0.8	0.8	0.8	Y	Lesser-of	No	Temporary	
Florida	0.49	0.48	0.53		Lesser-of	Partial	Temporary	
Georgia	0.7	0.68	0.89		Lesser-of	Partial	Temporary	
Hawaii	0.57	0.56	0.54	Y	Full	No	No/minimal	
Idaho	0.89	0.86	1		Lesser-of [c]	No	Extended	
Illinois	0.54	0.53	0.48	Y	Lesser-of	No	Temporary	4/1/2014
Indiana	0.55	0.53	0.75	Y	Lesser-of	No	Temporary	
Iowa	0.77	0.75	0.98	Y	Full	Full	No/minimal	
Kansas	0.82	0.79	0.73		Lesser-of	No	Temporary	
Kentucky	0.72	0.7	0.67		Lesser-of	No	Temporary	
Louisiana	0.75	0.69	0.67	[a]	Lesser-of	No	Temporary	
Maine	0.63	0.61	1	[a]	Full	Full	No/minimal	
Maryland	0.7	0.97	0.92	Y	Lesser-of	Partial [d]	Temporary	
Massachusetts	0.68	0.7	0.7	Y	Lesser-of	No	Temporary	10/1/2013
Michigan	0.46	0.44	0.71	Y [a]	Lesser-of	Partial	Temporary	3/1/2105
Minnesota	0.73	0.71	0.78	Y	Lesser-of	No	Temporary	
Mississippi	0.9	0.89	1		Full	Full	No/minimal	
Missouri	0.57	0.56	0.55		Full [e]	No	No/minimal	
Montana	0.94	1	1.06	[a]	Lesser-of	No	No/minimal	
Nebraska	0.76	0.75	1.01	[a]	Full	Full	No/minimal	
Nevada	0.68	0.66	0.95	Y	Lesser-of	Partial	Extended	
New Hampshire	0.6	0.58	0.56	Y [a]	Lesser-of	No	Temporary	
New Jersey	0.5	0.48	0.53	Y	Lesser-of	Partial	Temporary	
New Mexico	0.85	0.82	1	Y	Lesser-of	Full	Extended	
New York	0.42	0.48	0.44	Y	Partial [f]	No	Temporary	1/1/2015
North Carolina	0.85	0.8	0.79		Lesser-of	No	Temporary	
North Dakota	1.35	1.4	1	Y	Lesser-of	No	No/minimal	
Ohio	0.59	0.57	0.59	Y	Full	No	No/minimal	5/1/2014
Oklahoma	0.97	0.89	0.87		Full	No	No/minimal	
Oregon	0.72	0.73	0.77	Y	Lesser-of	Partial	Temporary	
Pennsylvania	0.56	0.52	0.51	[a]	Lesser-of	No	Temporary	
Rhode Island	0.33	0.32	0.33	Y	Lesser-of	No	Temporary	7/1/2016
South Carolina	0.74	0.72	1		Lesser-of	Full	Extended	2/1/2015
South Dakota	0.69	0.71	0.71		Full	No	No/minimal	

© 2021 Fung V et al. *JAMA Network Open.*

eTable. Summary of State Medicaid Policies (continued)

State	Medicaid to Medicare Fee Index			Medicaid Expansion as of 2014	Dual-eligible Medicare cost-sharing reimbursement policy as of 2015	Fee bump status as of 2015	State group for analyses	Dual demo enroll start date
	2012[1]	2014[2]	2016[3]					
Tennessee	*Excluded from analyses because TN does not have a fee-for-service Medicaid program*							
Texas	0.61	0.59	0.58		Lesser-of	No	Temporary	3/1/2015
Utah	0.74	0.74	0.86		Lesser-of	No	Temporary	
Vermont	0.81	0.8	0.84	**Y**	Full	Partial	No/minimal	
Virginia	0.74	0.73	0.84	[a]	Lesser-of	No	Temporary	
Washington	0.66	0.64	0.65	Y	Lesser-of	No	Temporary	
West Virginia	0.74	0.74	0.74	Y	Lesser-of	No	Temporary	
Wisconsin	0.6	0.58	0.48		Lesser-of	No	Temporary	
Wyoming	0.96	0.93	0.93		Lesser-of	No	No/minimal	

[a] AK expanded 9/1/15; LA expanded 7/1/16; ME adopted expansion but has not yet implemented; MI expanded 4/1/14; MT expanded 1/1/16; NE will hold referendum on expansion 11/18; NH expanded 8/15/14; PA expanded 1/1/15; VA will expand 1/1/19

[b] Despite Urban Institute-cited reduced funding, CT continued the fee bump in Jan. 2015. See http://www.huskyhealthct.org/providers/provider_postings/Primary_Care_Increased_Payments_Policy.pdf

[c] ID lesser-of policy excludes QMB-Only beneficiaries

[d] For Apr. to Jun. 2015, fees for evaluation and management procedures were reduced to 87% of Medicare. See https://mmcp.health.maryland.gov/Documents/JCRs/2016/physicianfeeJCRfinal1-17.pdf

[e] Sources disagreed; this figure pulled from MACPAC March 2013 Report to Congress. See https://www.macpac.gov/publication/march-2013-report-to-the-congress-on-medicaid-and-chip/

[f] Reimbursed 100% of Medicare deductible and 20% of Medicare coinsurance until July 2015, then lesser-of. See https://www.health.ny.gov/health_care/medicaid/program/update/2015/jun15_mu.pdf

© 2021 Fung V et al. *JAMA Network Open.*

eFigure 1. Changes in the percent of dual-eligible vs. non-dual-eligible beneficiaries with at least one annual visit with a primary care provider before and after the fee bump[a]

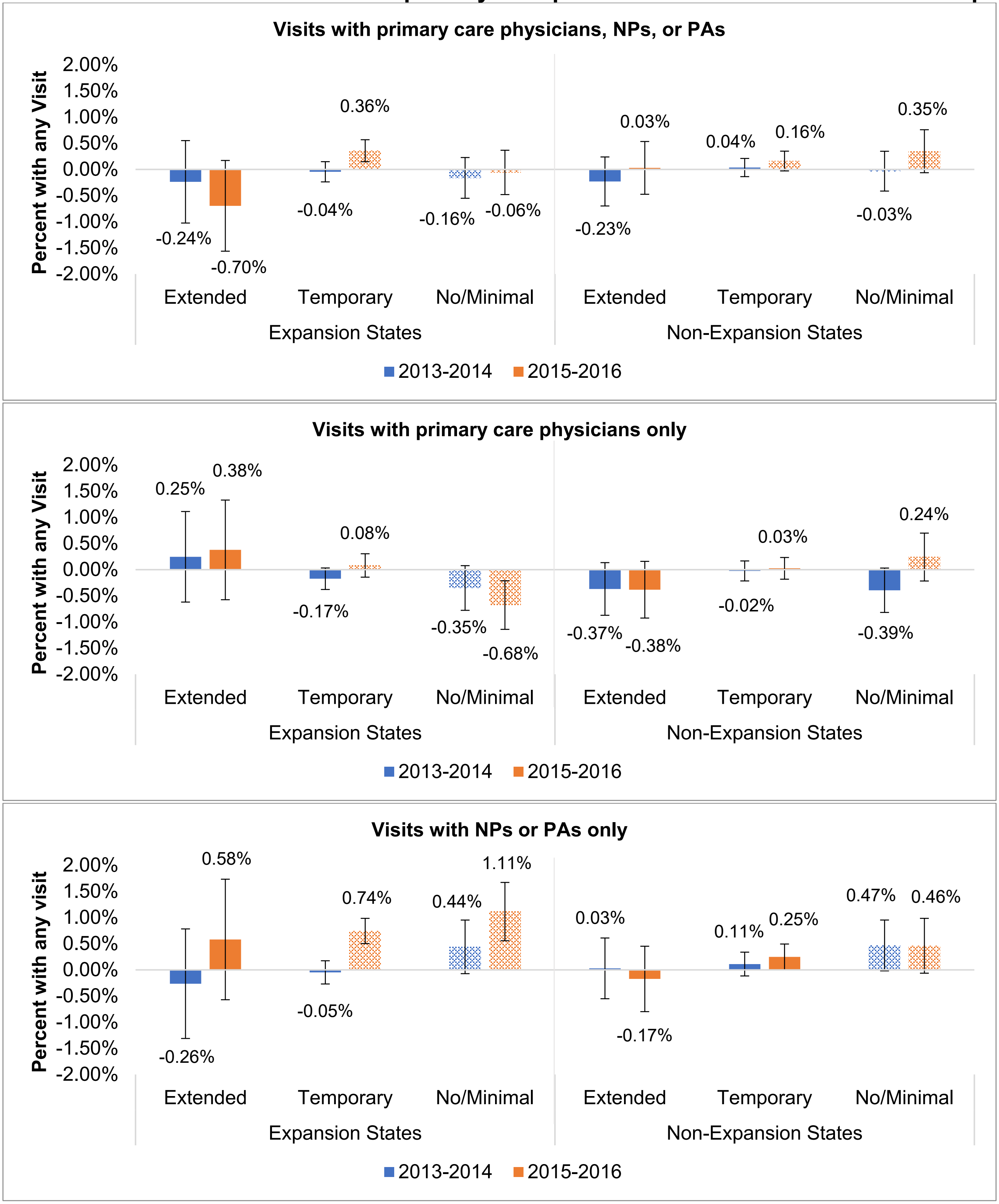

[a] Solid bars represent years in which the fee bump was active; hashed bars represent years in which the fee bump expired or there was no/minimal fee bump.

© 2021 Fung V et al. *JAMA Network Open.*

eFigure 2. Change in monthly visits to Federally Qualified Health Centers and Rural Health Centers for dual vs. non-dual beneficiaries before and after the fee bump[a]

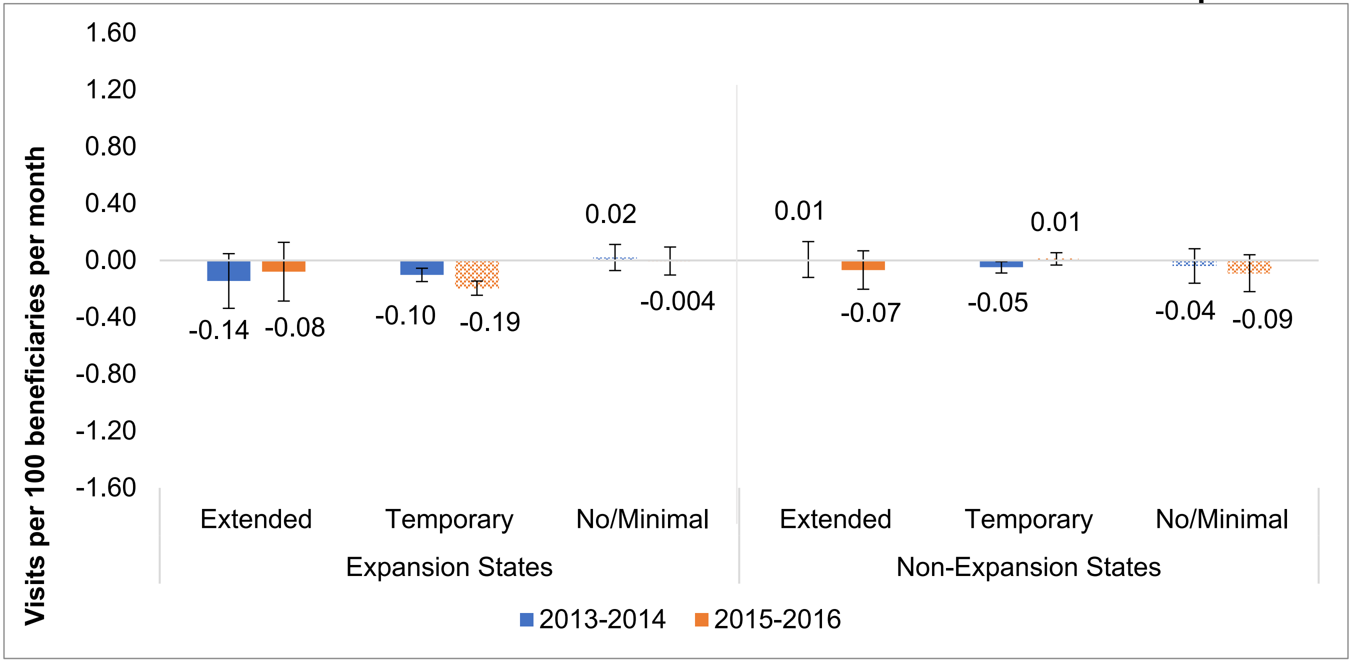

[a] Solid bars represent years in which the fee bump was active; hashed bars represent years in which the fee bump expired or there was no/minimal fee bump. Models also adjust for the % of residents in the county insured in each year, individual-level HCC scores in each year, an annual flag for ACO alignment, and state*year and state*month fixed effects.

© 2021 Fung V et al. *JAMA Network Open.*

eReferences

1. Kaiser Family Foundation. Medicaid-to-Medicare Fee Index. http://kff.org/medicaid/state-indicator/medicaid-to-medicare-fee-index/. Updated 2012. Accessed October 15, 2019.
2. Zuckerman S, Skopec L, McCormack K. *Reversing the Medicaid Fee Bump: How Much Could Medicaid Physician Fees for Primary Care Fall in 2015? Evidence from a 2014 Survey of Medicaid Physician Fees.* Urban Institute. 2014.
3. Kaiser Family Foundation. Medicaid-to-Medicare Fee Index. https://www.kff.org/medicaid/state-indicator/medicaid-to-medicare-fee-index/?currentTimeframe=0&sortModel=%7B%22colId%22:%22Location%22,%22sort%22:%22asc%22%7D. Accessed October 22, 2019.

© 2021 Fung V et al. *JAMA Network Open.*
